# Supplementary material for: Altered Gut Microbiota Patterns in Young Children with Recent Maltreatment Exposure
Source: Biomolecules. 2024 Oct 16;14(10):1313. doi: 10.3390/biom14101313 (PMC11506340; doi:10.3390/biom14101313)
Supplement: Supplementary file 1 [file biomolecules-14-01313-s001.zip › biomolecules-3095302-supplementary.pdf]

Supplementary materials

Supplementary Figure S1: Heatmap representing the relative abundance of the 15 most dominant genera in the no maltreatment (A), low maltreatment (B) and high maltreatment groups (C).

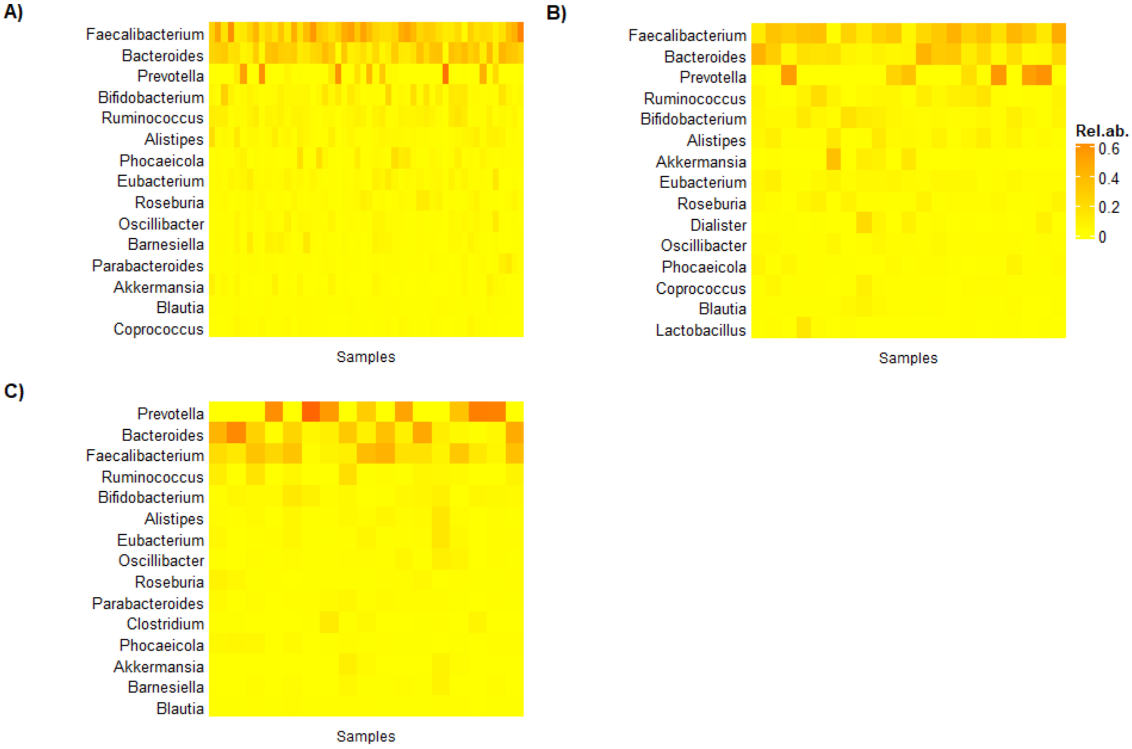

Supplementary Table S1: Comparison of gut microbial alpha diversity with interaction term of maltreatment exposure and CBCL total score as predictor, and BMI, SES, sex and antibiotic use as covariates. (a) Shannon index. (b) Simpson index.

**a. Shannon index (ANCOVA)**	**F (df)**	***p* value**
BMI	0.516 (77,1)	.475
Sex	3.075 (77,1)	.084
SES	0.446 (77,1)	.506
Antibiotic use	0.449 (77,1)	.505
Maltreatment exposure	0.202 (77,1)	.818
CBCL total score	0.018 (77,1)	.892
Maltreatment exposure * CBCL total score	0.012 (77,2)	.988
**b. Simpson index (ANCOVA)**	**F (df)**	***p* value**
BMI	1.189 (75,1)	.279
Sex	1.172 (75,1)	.282
SES	0.318 (75,1)	.575
Antibiotic use	0.044 (75,1)	.834
Maltreatment exposure	0.488 (75,1)	.616
CBCL total score	0.139 (75,1)	.710
Maltreatment exposure * CBCL total score	0.075 (75,2)	.928

Supplementary Table S2: PERMANOVA with the interaction term of maltreatment exposure and CBCL total score as a predictor, including BMI, sex, SES and antibiotic use as covariates.

**Gut microbial beta diversity**	**F (df)**	**Adj. *p* value**
BMI	1.387 (77,1)	.224
Sex	1.402 (77,1)	.232
SES	0.874 (77,1)	.480
Antibiotic use	2.142 (77,1)	.070
Maltreatment exposure * CBCL total score	0.789 (77,2)	.630
